# Supplementary material for: Examining Public Messaging on Influenza Vaccine over Social Media: Unsupervised Deep Learning of 235,261 Twitter Posts from 2017 to 2023
Source: Vaccines (Basel). 2023 Sep 24;11(10):1518. doi: 10.3390/vaccines11101518 (PMC10610639; doi:10.3390/vaccines11101518)
Supplement: Supplementary file 1 [file vaccines-11-01518-s001.zip › vaccines-2579232-supplementary.pdf]

Supplementary Material

Table S1. Top 10 tweets that have received much public attention for each topic

Sample Tweets with high public attention scores	Public attention score [1]
**TOPIC 1: PUBLICISING CAMPAIGNS TO ENCOURAGE INFLUENZA VACCINATION**	
1. ITALY: Nationwide quarantine SOUTH KOREA: Wide-scale testing USA: Turns out the Coronavirus is a lot like the flu. Would the flu vaccine help? No? Okay. Well, I heard its like the flu.	17615
2. BREAKING: Pfizer CEO Albert Bourla Announces At Davos New mRNA Flu Vaccine Due By June Or July This Year And A COVID/Flu Combination Vaccine Already In The Works "We started experiments to make one vaccine for both covid and flu."	12074
3. "Take care of your employees and they will take care of your business." Flu vaccine today	7667
4. How did they make the flu jab if we didn't have flu last year?	6175
5. It was less than 35,000 deaths over a longer period of time and there's a flu vaccine lol. Besides that, you're talking about globally less than 1% of people who died from the flu vs 3.4% with Covid-19. More than triple the deaths. Why do people still make this comparison?	4375
6. I don't recall anyone being called an "anti vaxxer" for not accepting the flu jab.	4114
7. Pencil in time for your familys FREE #FluShots #flu shot clinic! The clinic is open 10/16-10/17, 12-4pm Polar Bear parking lot, north of the parking garage on Illinois St. For more dates and details #FluVaccine #flushot	3771
8. New CDC study shows flu vaccine prevented more than 7 million flu illnesses, 109,000 flu hospitalizations & 8,000 flu deaths during 2017-18 #fluseason. Protect yourself & your family with a #flu vaccine today if you have not been vaccinated this season.	2234
9. There’s still time to get a flu vaccine. You can find flu vaccines near you on #FightFlu #PublicHealth	1198
10. Adults need vaccinations too. Even adults that are healthy need the annual influenza vaccine. Learn more about the impact that low #vaccination rates can have and why its important for people to get vaccinated. #NIAM #ivaxtoprotect	1014
**TOPIC 2: PUBLIC EDUCATION ON THE SAFETY OF INFLUENZA VACCINE DURING PREGNANCY**	
1. FACT: Pregnant women should especially get the flu vaccine since their immune systems are weaker than usual. The inactivated flu vaccine is safe at any stage of pregnancy	353
2. Did you know there is an association between spontaneous abortion and the inactivated flu vaccine containing H1N1? #flu #influenza #informedconsent #picphysicians #vaccines	202
3. By taking the vaccine, Im trying to reassure my pregnant women of how safe it is to be vaccinated. Maternity matron Alice had her COVID-19 booster and flu vaccines. If you're eligible, book your flu and COVID-19 booster vaccines now.	196
4. Not enough pregnant women are getting the flu vaccine and it's putting themselves, their babies and the public at risk, according to a new CDC report.	156
5. MYTH: The flu vaccine might hurt my baby. If youre pregnant, the flu vaccine doesnt harm your unborn baby. In fact it can protect your baby from flu for the first few months of life. Find out more:	155
6. Women who are or will be pregnant during flu season should get the flu vaccine. All pregnant women should receive a Tetanus, Diphtheria, & Pertussis vaccination during each pregnancy, as early in the 27-36-weeks-of-gestation window as possible.	149
7. Hey Lindsay these are PRRs - proportional reporting ratios. That is, reports to VAERS of this vaccine compared to the influenza vaccine. That's different from a "57-fold increase in miscarriages"! The base rate for miscarriages is 5% btw.	135
8. Mothers who receive the influenza vaccine during pregnancy can reduce the chance their infant has the flu by 1/3. #WhyIVaccinate #VaccinesWork	134
9. #FluFactFriday: Changes in the immune system make pregnant women more likely to experience severe illness from #flu. If you are pregnant, get your flu vaccine now for the best protection for you and your baby. #FightFlu	123
10. Also (this is purely anecdotal): pregnant patients whove had only 2 doses of vaccine get as sick as those who are unvaccinated. A vaccine given last year isnt going to protect you, just like last years influenza vaccine isnt going to protect you from severe flu complications	117
**TOPIC 3: PUBLIC EDUCATION ON THE APPROPRIATE AGE TO RECEIVE INFLUENZA VACCINE**	

1.	#Flu can be serious and can cause severe illness. CDC recommends that everyone 6 months and older get a flu vaccine. There's still time to get yours if you haven't already.	1962
2.	Dr. Fauci: "We're encouraging everyone six months of age or older, so really everyone, to get a flu vaccine."	1818
3.	Dr. Anthony Fauci: "We're encouraging everyone 6 months of age or older, so really everyone, to get a flu vaccine."	884
4.	The recommends that all adults and children older than six months receive the flu vaccine by the end of October.	455
5.	released updated flu vaccine recommendations for 20212022. #COVID19 vaccines may be given with #flu vaccines. Everyone 6 months and older should get a #fluvaccine this fall.	449
6.	#HappyHalloween! This year, enjoy the candy and costumes, but dont forget a #flu vaccine! CDC recommends that everyone 6 months and older get a flu vaccine NOW for the best protection. Learn more: #FightFlu	445
7.	To protect children amid #COVID19, it's vital that kids receive routine vaccinations, including the flu vaccine, reports. Dr. Sally Goza: "What we don't need is an outbreak of measles or whooping cough or the flu come fall." #VaccinesWork	318
8.	Children older than 6 months should receive their flu vaccine as soon as it is available, reports. With #COVID19 also circulating, it is the best way to reduce the risk of children suffering severe illness this season. #VaccinesWork	279
9.	Who should get a #fluvaccine? Everyone who is 6 months of age and older. Getting #fluvax can reduce flu illnesses, missed work days, and even flu-related hospitalizations. Get a flu vax and do your part to help #fightflu.	197
10.	Its time to start thinking about #flu vaccines the best way to protect against the worst effects of flu. CDC recommends everyone 6 months & older get an annual flu vaccine by the end of October.	182
**TOPIC 4: PUBLIC EDUCATION ON THE IMPORTANCE OF INFLUENZA VACCINE DURING PREGNANCY**		
1.	Pregnant people are at higher risk of serious flu illness, but fewer pregnant people are getting a #flu vaccine in recent seasons, with the lowest coverage among Black, non-Hispanic pregnant people.	161
2.	We are rapidly approaching flu season and this illness can be very serious for pregnant women and their babies. Dr Vanessa Mackay, Consultant Obstetrician and Gynaecologist, explains why we recommend pregnant women have the flu vaccine, which is available to them now.	159
3.	As the flu season is looming & we are all probably feeling a bit nervous about whats to come & what the powers that be have in store for us, here is some info about the flu jab with references. My mum almost died & my friend miscarried at 7 months. Inform your loved ones.	155
4.	Drop-in clinic's will be held for pregnant women in Princess Royal Maternity, 4:30 to 8:30pm Wed 22nd & Thur 23rd December. Along with your partner get 1st, 2nd or booster vaccination. Also get flu vaccine at the same time.	123
5.	The #COVID19 vaccine is the best way to protect pregnant people and fetuses from severe illness, preterm birth, and stillbirth. Its safe to get the primary series and boosters in any trimester and at the same time as the flu vaccine.	102
6.	Dr spoke about the flu vaccine with the flu jab is the best protection from flu and its free for pregnant women. Just ask your pharmacist, GP or midwife. #GetYourFluJab #Flu	99
7.	Sometimes illness can be more than just a common cold. Protect your #baby with the #flu vaccine. #NIVW	98
8.	Vaccines are safe and important for people who are pregnant. Erica Adams got her flu vaccine today from Pharmacist Shelley Russell at Lawtons to protect herself, her baby, and the people she cares about. #getaflushot	87
9.	Pregnant women are eligible for a free flu jab and an autumn booster of the COVID-19 vaccine. We and continue to urge all pregnant women and people to have both vaccines as we approach winter:	84
10.	Flu jab time! Pregnant women can suffer severe complications if they contract the flu so here I am, Director of Midwifery , getting my flu jab to protect the women under our care and our staff #getthatjab	81

[1] Public attention score for each tweet was calculated as the sum of the retweet count, reply count, like count and quote count
